# Alternation as a form of allocation for quality improvement studies in primary healthcare settings: the *on-off* study design

**DOI:** 10.1186/s13063-015-0904-x

**Published:** 2015-08-25

**Authors:** Nonsikelelo Mathe, Steven T. Johnson, Lisa A Wozniak, Sumit R. Majumdar, Jeffrey A. Johnson

**Affiliations:** Alliance for Canadian Health Outcomes Research in Diabetes, School of Public Health, University of Alberta, 2-040 Li Ka Shing Center for Health Research Innovation, Edmonton, AB T6G 2E1 Canada; Center for Nursing and Health Studies, Faculty of Health Disciplines, Athabasca University, Athabasca, AB Canada; Alberta Diabetes Institute, Edmonton, AB Canada; Department of Medicine, Faculty of Medicine and Dentistry, University of Alberta, Edmonton, AB Canada

**Keywords:** Alternation, Controlled trials, Quality improvement, Primary healthcare

## Abstract

**Background:**

Randomized controlled trials are considered the “gold standard” for scientific rigor in the assessment of benefits and harms of interventions in healthcare. They may not always be feasible, however, when evaluating quality improvement interventions in real-world healthcare settings. Non-randomized controlled trials (NCTs) are designed to answer questions of effectiveness of interventions in routine clinical practice to inform a decision or process. The *on-off* NCT design is a relatively new design where participant allocation is by alternation. In alternation, eligible patients are allocated to the intervention “*on*” or control “*off* ” groups in time series dependent sequential clusters.

**Methods:**

We used two quality improvement studies undertaken in a Canadian primary care setting to illustrate the features of the *on-off* design. We also explored the perceptions and experiences of healthcare providers tasked with implementing the *on-off* study design.

**Results and discussion:**

The *on-off* design successfully allocated patients to intervention and control groups. Imbalances between baseline variables were attributed to chance, with no detectable biases. However, healthcare providers’ perspectives and experiences with the design in practice reveal some conflict. Specifically, providers described the process of allocating patients to the *off* group as unethical and immoral, feeling it was in direct conflict with their professional principle of providing care for all. The degree of dissatisfaction seemed exacerbated by: 1) the patient population involved (e.g., patient population viewed as high-risk (e.g., depressed or suicidal)), 2) conducting assessments without taking action (e.g., administering the PHQ-9 and not acting on the results), and 3) the (non-blinded) allocation process.

**Conclusions:**

Alternation, as in the *on-off* design, is a credible form of allocation. The conflict reported by healthcare providers in implementing the design, while not unique to the *on-off* design, may be alleviated by greater emphasis on the purpose of the research and having research assistants allocate patients and collect data instead of the healthcare providers implementing the trial. In addition, consultation with front-line staff implementing the trials with an *on-off* design on appropriateness to the setting (e.g., alignment with professional values and the patient population served) may be beneficial.

**Trial registration:**

Health Eating and Active Living with Diabetes: ClinicalTrials.gov identifier: NCT00991380 Date registered: 7 October 2009.

Controlled trial of a collaborative primary care team model for patients with diabetes and depression: Clintrials.gov Identifier: NCT01328639 Date registered: 30 March 2011.

## Background

The development and evaluation of complex interventions in healthcare requires study designs that are methodologically sound, but which also acknowledge and adapt to the complex local context of healthcare delivery [1, 2], including existing professional culture and values. Among the hierarchy of study designs, randomized controlled trials (RCTs) are generally considered the “gold standard” with respect to scientific rigor [1]. However, issues of applicability, feasibility and context are often lost when RCTs are conducted and reported. As Schwartz and Lellouch [3] suggested in the late 1960s and more recently echoed by Tunis [4], trials that serve to generate knowledge for decision-makers must differ from the prototypical RCT. These non-randomized controlled trials (NCTs) are designed to answer questions of effectiveness of interventions in routine clinical practice [5].

One challenge often faced by investigators when undertaking NCTs is reluctance by healthcare providers and organizations to use random allocation [6]. Thus, various alternatives to randomized designs have been developed and used to evaluate healthcare interventions [6, 7]. Historically, and prior to the advent of randomized trials in the 1940s, alternation was a common form of patient allocation to treatment comparison groups [8, 9]. The *on-off* design is an example of a NCT study design based on the concept of alternation, but which has not been widely described in the literature. The intent of this article is to describe the features of the *on-off* design, using two recent trials as examples. In addition, we explored the perceptions and experiences of healthcare providers tasked with implementing the *on-off* study design in their primary care settings. Therefore, our intent is to describe the *on-off* design, discussing potential strengths and weaknesses and its appropriateness to end users (i.e., healthcare providers and policy decisionmakers) of quality improvement research.

### Features of the *on-off* design

Regardless of the setting, the common and distinguishing feature of the *on-off* design is the alternating allocation to intervention or control groups based on the date or time of patient presentation or identification. As with randomization, the intent of alternation is to ensure treatment comparisons are made in patients with as similar characteristics as possible [8, 9]. Eligible participants from a recruitment pool (or defined population) are allocated to intervention or control groups in sequential clusters. The initial allocation of the clusters can be determined at random, followed by a second equal recruitment period to the alternate exposure, which has been reported as a randomized crossover cluster design [2, 10, 11]. This crossover recruitment to clusters may be repeated on an alternating defined time frame (e.g., 1 month), until the required sample size is obtained or the time for identification expires. In some cases the determination of the initial order of exposure may be non-random, followed by repeated, alternating recruitment, which may be described as alternation. This method has been previously used in quality improvement studies [12–14], and meets study design quality criteria for controlled trials to permit inclusion in the Cochrane Collaborations’ Effective Practice and Organization of Care (EPOC) systematic reviews [15].

Thus, the main strengths of the *on-off* design are its ability to allocate patients to comparison groups efficiently, while maintaining compatibility with real-world practice settings. That is, the study should not interfere with routine functioning of a particular primary care environment and where local staff is involved in the implementation [4]. In addition, the *on-off* design balances case mix and controls for Hawthorne or volunteer effects, controls for temporal (secular) trends [16] as well as issues related to institutional learning, while also avoiding “Zelen’s” design [17, 18], which many consider to be unethical (randomizing patients prior to obtaining consent) and demonstrates external validity in terms of implementing successfully at multiple sites.

One of the major advantages of randomization is providing the theoretical background for the statistical comparison of outcomes in a trial [19]. The other is the likelihood of achieving balance in known and unknown confounders between treatment comparison groups at baseline. Alternation is intended to provide balanced groups, but this may be threatened due to lack of concealment and blinding. By alternating blocked time periods, alternation may reduce the threat of unbalanced groups. A related threat subsequent to the initial allocation is the potential for contamination between intervention and control arms. As with any controlled trial, for the *on-off* design to minimize threats to internal validity, a prior belief of equipoise must exist; that is, it is assumed there is genuine uncertainty about the benefits of the intervention over usual care.

Because alternating allocation is predictable, concealment is difficult and healthcare providers may selectively enroll participants in the intervention or control arms. Such a selection bias would threaten internal validity of the study. The direction of such a bias might not be predictable, however, and would depend on the front-line healthcare provider beliefs of the effectiveness of the intervention under study. For example, providers may feel a strong moral or professional obligation to not allocate patients to “usual care” when the alternative is a new treatment strategy they believe to be more effective. On the other hand, providers may also preferentially recruit healthier patients into the intervention arm if they believe that this might lead to a conclusion in the study of better outcomes with the intervention.

## Methods

### Two examples of trials using *on-off* design

We recently undertook two studies that used the *on-off* design. Our trials were conducted in four primary care networks (PCN) in Alberta, Canada as part of Alberta’s Caring for Diabetes (ABCD) Project [20, 21]. Both trials received ethical approval from the University of Alberta’s Health Research Review Board.

#### Trial 1. Controlled trial of a collaborative primary care team model for patients with diabetes and depression (TeamCare)

**Fig. 1:**
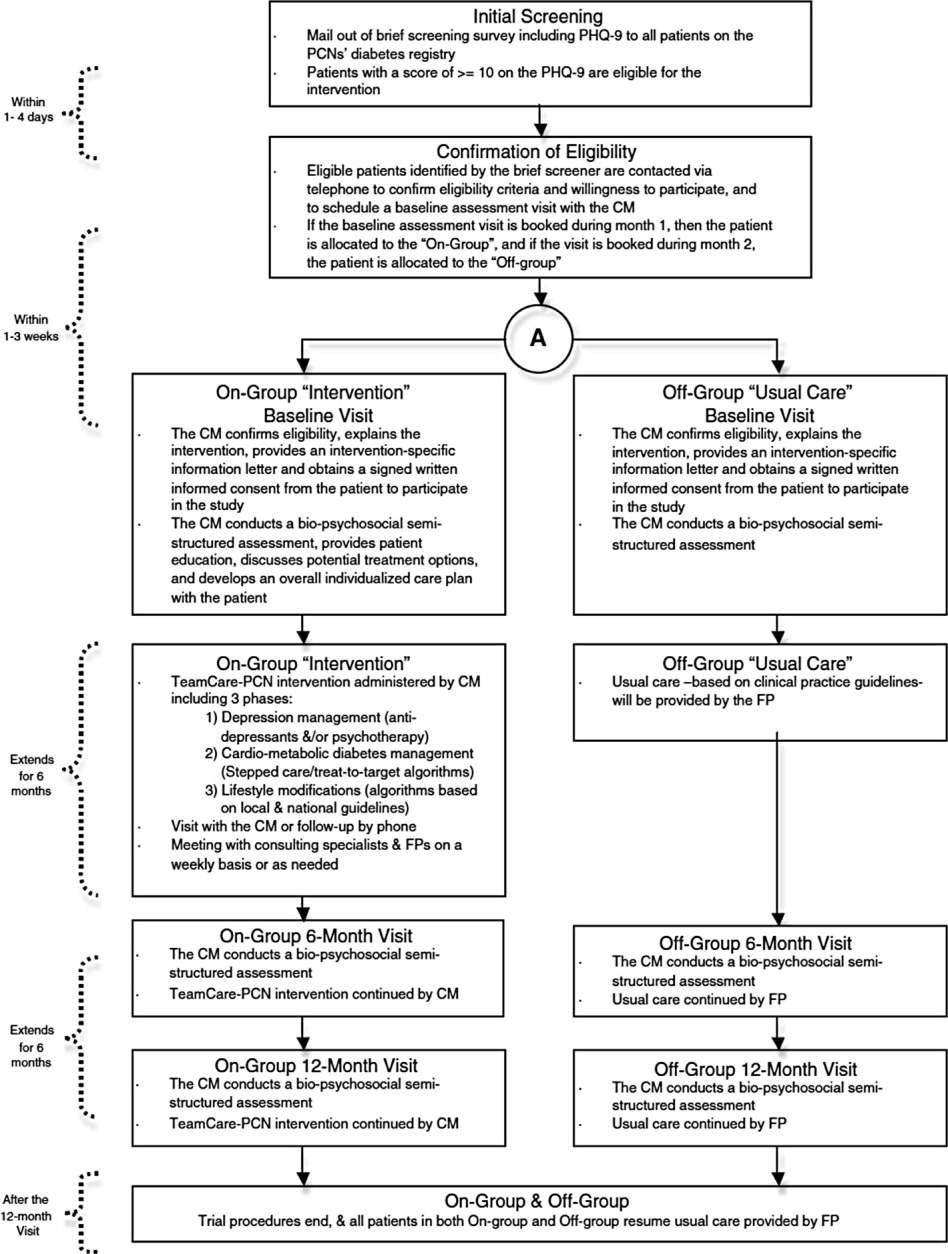
Schematic of TeamCare [10]

The aim of this intervention was to evaluate a collaborative care model for patients with comorbid type 2 diabetes and depression within established PCNs (Fig. 1) [20]. The TeamCare intervention involved a registered nurse care manager (CM), who coordinated the collaborative management for patients with diabetes and depression. Patients with type 2 diabetes registered at a participating PCN (under family physician (FP) care and on a patient registry) and scoring ≥ 10 on the Patient Health Questionnaire 9-item (PHQ-9), a brief depression screening survey, were eligible for the intervention. All eligible and consenting participants were allocated to the intervention (*on*) or active-control (*off*) arms using a monthly time-series *on-off* design as follows: participants who screened positive were contacted by the CM to book an initial assessment. Participants who were booked for their initial assessment during month 1 were allocated to the intervention arm (*on*); those who were booked in month 2 were allocated to the active-control arm (*off*). Participants were assessed at baseline, 6 and 12 months.

#### Trial 2. Healthy eating and active living for diabetes in primary care networks (HEALD)

**Fig. 2:**
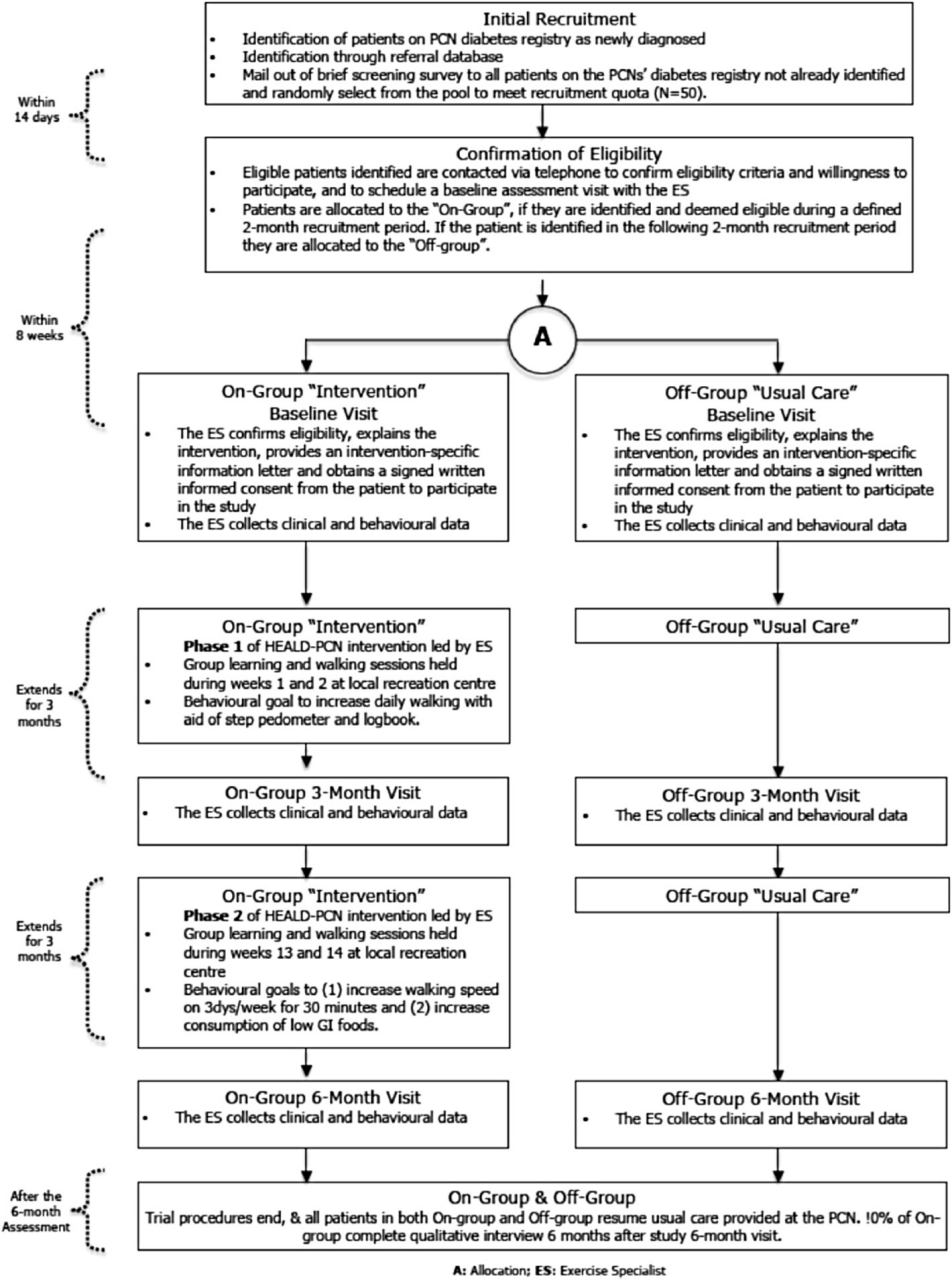
Schematic of HEALD [11]

The aim of this intervention was to evaluate the implementation of an evidence-based self-management program for patients with type 2 diabetes, within established PCNs (Fig. 2) [21]. The HEALD intervention was based on a previously pilot-tested self-management program in this patient population [22]. It consisted of 2 phases, lasting a total of 24 weeks, with emphasis on physical activity (walking) and nutritional elements. Potentially eligible patients were identified and recruited through a patient registry, developed as part of the PCN’s chronic disease management structure. Eligible patients were contacted via telephone to establish willingness to participate and a baseline assessment with an exercise specialist was scheduled. A recruitment cluster was filled through a number of strategies, with an aim of accruing 50 potentially eligible participants within a defined time frame. The first strategy was through active identification of newly diagnosed patients on each of the PCN’s diabetes patient registries. Patients were allocated to the *on* group if they were deemed eligible during a defined 2-month period and they would enter the study on a predefined date. Similarly, recruitment and allocation to the *off* group occurred over the subsequent 2 months.

### Quantitative analysis

To assess allocation balance between *on* and *off* groups in the TeamCare and HEALD trials, baseline characteristics were compared. Ordinarily, it is unnecessary to test for differences in participant characteristics of a controlled trial [23]; however, in this case it is advisable to test if the process of allocation is hypothesized to have led to bias [23, 24]. We used the Student’s *t* test to identify differences in age, systolic and diastolic blood pressure, glycosylated hemoglobin (HbA1c), low-density lipoprotein (LDL) cholesterol and high-density lipoprotein (HDL) cholesterol, triglycerides, total cholesterol, body mass index (BMI) and waist circumference for both TeamCare and HEALD. In addition, depression (PHQ-9) scores were assessed for TeamCare only and weight, resting heart rate, 3-day pedometer steps, and indicators of dietary intake assessed in HEALD only. Differences were compared from both an absolute and statistical perspective. We considered clinically important differences when known or recognized for clinical variables. Randomness of the allocation of participations was tested, based on the set of *p* values for the comparisons of baseline characteristics of both studies, by computing the goodness of fit (GOF) chi-square statistics relative to a uniform distribution. Statistical significance was considered at a level of < 0.05. All analyses were performed using STATA SE 10.1, StataCorp, College Station, TX, USA.

### Qualitative data collection and analysis

We identified the experiences and perceptions of the PCN staff (i.e. executive directors (ED), chronic disease managers (CDM), care managers (CM), and exercise specialists (ES)), involved in implementing the trials across the four PCNs that arose during semi-structured interviews about the interventions. We conducted interviews at multiple times (i.e., baseline, midpoint, and post-intervention). Interviews were digitally recorded, transcribed verbatim by an independent transcriptionist and verified for accuracy. In addition to interviews, we documented minutes of meetings and field notes. All qualitative data sources, including interview transcripts and notes were compiled in and managed using Nvivo 10 software (QSR International, Burlington, MA, USA). Content analysis [25] was used to identify emerging codes using a general inductive approach [26]. Throughout the data analysis process, we discussed emerging codes and concepts during formal research team meetings. Any discrepancies were discussed until consensus was reached. We used study codes for highlighted quotes to ensure participant confidentiality across the PCNs, using the following respondent group categorizations: “PCN management” refers to the ED or CDM and “PCN provider” refers to the CM or exercise specialists involved in implementing TeamCare or HEALD.

## Results and discussion

### Our perceptions and experiences with the *on-off* design as researchers

As recruitment and allocation of patients proceeded in our two quality improvement trials in primary care, we evaluated the balance of patient characteristics. In TeamCare, we were initially concerned that the allocation of patients may have been biased, as we achieved an imbalance in numbers (*n* = 95 for intervention and *n* = 62 for control). However, there were no clinically important differences in baseline characteristics between the intervention and active-control groups in all variables (Table 1). It was particularly important that the PHQ-9 score, the main outcome variable, was in fact balanced between groups at baseline. Thus, despite differences in the number of patients allocated to each arm, it did not appear that the CM had introduced a selection bias in the characteristics of the patients. For Table 1, the GOF test X2 (10) = 10.1134 with a tail probability of 0.43 suggests that these *p* values are not detectably different from a uniform distribution, suggesting the 2 groups were essentially randomly allocated.

**Table 1:** Baseline characteristics of TeamCare

Characteristic	Intervention (*n* = 95)	Active control (*n* = 62)	
	*n* or mean (% or (SD)	*n* or mean (% or SD)	*p* value^a^
Age (years)	57.35 (9.97)	59.18 (8.55)	0.2
Sex (% female)	58 (61 %)	29 (47 %)	0.08
BMI (kg/m^2^)	37.07 (8.10)	36.43 (8.19)	0.6
Waist circumference (cm)	114.0 (18.55)	115.7 (14.50)	0.5
PHQ-9	14.47 (3.85)	14.59 (3.47)	0.9
HbA1_c_ (%)	7.4 (1.78)	7.8 (1.70)	0.2
Systolic BP (mmHg)	126.59 (15.46)	123.54 (16.63)	0.3
Diastolic BP (mmHg)	75.98 (9.43)	74.59 (8.22)	0.4
LDL cholesterol (mmol/L)	2.22 (0.86)	2.15 (0.77)	0.6
HDL cholesterol (mmol/L)	1.09 (0.41)	1.04 (0.36)	0.4
Total cholesterol (mmol/L)	4.38 (1.11)	4.52 (0.98)	0.4
Education to less than high school level	11 (12 %)	10 (16 %)	0.4
Employed	48 (52 %)	31 (51 %)	1.0
White	81 (87 %)	49 (80 %)	0.1
Income (CAD)			
< $40,000	28 (30 %)	16 (26 %)	0.2
$40,000–$80,000	23 (25 %)	16 (26 %)	
> $80,000	26 (28 %)	13 (21 %)	
Refuse to answer	3 (3 %)	8 (13 %)	
Smoking	23 (25 %)	15 (25 %)	0.5
Alcohol use	70 (75 %)	41 (67 %)	0.3
Psychoactive medications^b^	46 (48 %)	27 (44 %)	0.6

^a^From *t* test for continuous data or chi-square for categorical data

^b^Antidepressants, anxiolytics, mood stabilizers, or antipsychotics

*BMI* body mass index, *BP* blood pressure, *HbA1c* glycosylated hemoglobin,* HDL* high-density lipoprotein*, LDL* low-density lipoprotein, *PHQ-9* Patient Health Questionnaire 9-item CAD=Canadian Dollars

In HEALD, a similar number of patients were allocated to the intervention and control arms. There was no significant difference between groups for the main study outcome variable of interest, which was the mean of 3-day total step count measured by pedometer (Table 2). There were significant differences in 2 baseline characteristics; intervention patients were 3 years younger than control patients (58 versus 61 years; *p* = 0.007) and BMI was different by 2 points (*p* = 0.04) although both mean values were in the obese category (Table 2). The differences noted in the HEALD participants’ baseline characteristics may not be clinically important, but may be indicative of a bias. The GOF test X2 (10) = 35.0493, with a tail probability of 0.0001, suggests that the *p* values are detectably different from a uniform distribution, implying the allocation did not randomly distribute these characteristics. However, there is still a possibility that these results are due to chance, given that ESs involved in implementing HEALD were unaware of the baseline 3-day step count and hence could not influence allocation based on this outcome measurement.

**Table 2:** HEALD baseline characteristics

Characteristic	Intervention (n =102)	Active control (*n* = 96)	
	*n* or mean (% or SD)	*n* or mean (% or SD)	*p* value
Age (years)	58.0 (8.2)	61.3 (8.4)	0.007
Sex (% female)	46 (48 %)	54 (54 %)	0.4
BMI (kg/m^2^)	34.6 (6.5)	32.5 (6.5)	0.04
Waist circumference (cm)	112.5 (14.9)	108.7 (15.4)	0.09
Weight (kg)	98.7 (20.6)	93.6 (20.8)	0.1
Resting heart rate (beats/minute)	71.0 (10.9)	70.7 (10.9)	0.8
3-day pedometer steps	16,761 (9238)	19,075 (10,096)	0.1
Glycemic index	52.3 (4.6)	51.1 (4.6)	0.1
Glycemic load	61.8 (15.3)	57.7 (16.6)	0.1
Energy intake (kcals)^a^	1318 (452)	1252 (467)	0.3
Systolic BP (mmHg)	125.8 (16.6)	125.4 (15.9)	0.8
Diastolic BP (mmHg)	75.0 (8.7)	76.9 (8.8)	0.1
HbA1c (%)	6.9 (1.2)	6.6 (0.9)	0.1
LDL cholesterol (mmol/L)	2.2 (0.7)	2.4 (0.9)	0.1
HDL cholesterol (mmol/L)	1.2 (0.4)	1.20 (0.5)	0.7
Total cholesterol (mmol/L)	4.2 (0.9)	4.5 (1.0)	0.1
Education to less than high school level	2 (1.9 %)	4 (4.2 %)	1.0
Employed	67 (65.7 %)	55 (57.3 %)	0.6
White	84 (82.3 %)	78 (81.2 %)	1.0
Income (CAD)			
< $40,000	26 (25.5 %)	17 (17.7 %)	0.8
$40,000–$80,000	39 (38.2 %)	58 (50 %)	
> $80,000	37 (36.3 %)	31 (32.3 %)	

^a^Energy in kilocalories estimated from food frequency questionnaires

*BMI* body mass index, BP blood pressure, *HbA1*_*c*_, glycosylated hemoglobin, *HDL* high-density lipoprotein, *LDL* low-density lipoprotein, CAD=Canadian Dollars

### Healthcare providers’ experiences and perceptions of the *on-off* design

The potential limitations with the *on-off* design in PCN depend largely on the perceptions of the front-line healthcare providers implementing them.

At baseline, PCN staff did not express concerns implementing the *on-off* design in the primary care setting. However, during and post intervention, PCN staff reported discomfort employing the design, and in the extreme described it as “unethical or immoral,” particularly in relation to TeamCare. The discomfort arose largely from a sense of conflict in professional principles and the commitment to provide care. The allocation to an “*off* ” group of patients in need of care seemed to conflict with the healthcare providers’ professional commitment to provide care to all, especially for those patients perceived as most in need. Further, while PCN staff recognized the value of research, they perceived this design as inappropriate for this stage of research.

The following excerpts demonstrate the perspectives of PCN staff in implementing the *on-off* design in a primary care setting.

#### Discomfort with the on-off design

PCN staff reported discomfort with the *on-off* design once they implemented it in practice in the primary care setting. More specifically, they viewed the design as “unethical” or “immoral” in relation to the TeamCare trial. In addition, PCN staff commented that the *on-off* design was not appropriate for this stage of research:

*I think we would have had a lot more buy-in since this is already a protocol that has been proven in other places to have worked. We’re not necessarily testing the protocol so much as the applicability to this population and, honestly, is there that much difference between Canadians and Americans as far as whether or not a protocol would work? (PCN provider)*

*(The) On-Off has been one of the major challenges. And I know in a pure research laboratory [using], very scientific methods, it makes abundant sense to do that because that is the “gold standard.” But in reality, I think that’s why you see so few people-related studies that are having an On-Off group unless it’s like a pharmaceutical double-blind. (PCN management)*

#### Conflict with professional commitment to provide care

PCN staff described the awareness of patients’ allocation status as causing conflict with professional training and experience of usual care:

*In regard to the model of how the study is structured, I think the way that it’s not blinded – and I don’t know how else to look at the study. I have some research experience. But having an unblinded study where there’s the On group and the Off group and then this Off group doesn’t really get anything from us other than having to fill out questionnaires. (PCN provider)*

PCN staff commented that it was difficult for healthcare providers to refer *off* patients in the TeamCare intervention to usual care either because patients or their physicians may not access care or because of patient safety and liability concerns:

*And is the client gonna really go back to the doctor? I have no control over that. He may say, “Yeah I’ll go,” but whether he actually goes? I’m not following him up in 2 weeks and saying, “Hey, did you get over to your doctor yet? (PCN provider)*

*Patient safety should, in my mind as a clinician, have to come [first]. And it didn’t feel like it was. And that’s hard to do as a nurse. Especially as the legal expectation for myself that I would not compromise in any other setting. And I didn’t feel I did compromise because I felt trust in my existing team. But if we didn’t have the mental health team in place in order to refer somebody to, I would have felt that I was neglecting patient care. (PCN provider)*

It was difficult for health service providers to refer *off* patients to usual care in cases where patients exhibited severe symptoms, or were struggling to manage their condition:

*This fellow yesterday, just as an example, he was 21(on the PHQ)… his A1c was 11.1, his lipids were all elevated, blood pressure. And then I had to say, “Hmmm. Well thanks for coming out.” So that feels unethical to me somehow ‘cause we know we could offer something more. So I have a hard time with that. This fellow yesterday, he said, “I feel like my wife and I are falling through cracks,” and here he gave a hand out and I had to say, “Well thanks for coming out. Go back to your doctor” – you know? Just didn’t seem right somehow. Can we make him in my On group? Could I switch him? And then when he says things like, “I feel like my wife and I have fallen through the cracks,” it’s like oh here’s another one, you’re falling through the cracks ‘cause – he’s so depressed, he feels like he’s on a treadmill. He feels like giving up. He feels like jumping off a bridge. So now I have to say, “Hey wait a minute, go back to your doctor,” rather than saying, “Hey, why don’t you come back next week and see me and we’ll talk some more about this.” (PCN provider)*

*So that way you wouldn’t have this group of people that you feel some of them have been the people that needed it the worst, you know? Looking at their PHQ scores and talking with them even just a brief amount of time that you talk. Actually some of them I’ve spent quite a bit of time talking to because they’re a 20 (on the PHQ). You can’t really offer them everything. You think, “Oh I’d really like to follow up this individual,” and you can’t do it. So it’s kind of annoying – not right. Morally not right for them. So that’s one thing I would change. (PCN provider)*

#### Support for research and suggestions for improving or altering study design

PCN staff reported valuing research; for example, when asked why they agreed to partner with the university, administrators stated that their PCN supports research in the primary care setting. In addition, administrators intended to use the study results to inform decision-making.

Several PCN staff commented on the need for control groups:

*I think the whole On/Off, having the two groups and still seeing them in the same setting or context of a PCN. We realize you have to have a control but that was tough. Because you can’t really offer a whole lot to the people who are really struggling because of that Off group. It’s hard to kind of design. I don’t know if that was something that could be improved or worked on.’Cause you do have to have a control group somewhat. (PCN provider)*

*One of the challenges is the On and Off group idea. ‘Cause obviously it’s been really hard for the nurses to say, “Well, you’re demonstrating all these symptoms and maybe you’re not managing so well with your diabetes but you can’t be part of the program.” And so that’s been a struggle for us, for sure. Obviously a necessity in terms of how the project works and what we need the outcomes to be. But yeah, definitely struggling with that. (PCN provider)*

PCN staff acknowledged the advantage of control groups in clinical trials, but described limited usefulness in primary care settings. In addition, having health service providers interact with *off*-group patients to collect data clashes with their training to provide care, problem-solve and offer services:

*See, it may be easier if somebody is used to research, this is just the way it goes. So you pull somebody in who’s a primary caregiver and put them into a position where they’re used to solving problems, they’re used to making suggestions. That’s what you do and then you have to take that away from them. It’s like you strip that ability away from them it’s like, “Okay, yeah.” (PCN management)*

Due to the challenges associated with lack of allocation concealment and the conflicting principles noted above, PCN staff recommended modifying the study design:

*As far as ongoing challenges, I still think that the Off group and the design methodology has been a major barrier to success. And in the future, I have a very strong recommendation that that design methodology be reconsidered. (PCN management)*

- Intervention/*on-*group patients can act as their own controls using their past histories:

*I’m not sure that you’re going to get better data or better results by having had this group of people that didn’t get the protocol. They could have been their own control group and then look at how many more people we could have offered it to. (PCN management)*

- Carefully match patients:

*I think people can be their own controls or you can carefully match. (PCN management)*

- Focus on qualitative rather than quantitative evidence:

*I know there’s other ways of doing research. I don’t know if you want it to be more qualitative than quantitative. (PCN provider)*

It is important to note that we modified the TeamCare protocol to allow *off-*group patients to cross over into the *on* group once they completed the *off* group cycle and if they were still experiencing depressive symptoms as one strategy to alleviate PCN staff’s concerns with the design.

When the efficacy of an intervention has been established and questions of internal validity have been adequately addressed, assessing effectiveness in a real-world setting is a logical next step. The most appealing aspect of NCTs is that they aim to achieve a balance between internal validity while including design elements that enhance external generalizability [27]. In many cases, randomization is a strength of a controlled trial. However, random allocation of patients, as seen in classic RCTs, may not be acceptable in some cases, such as where front-line healthcare providers object to participation in RCT designs, and alternative forms of allocation can be implemented, such as the *on-off* design described here.

Using the examples of the TeamCare and HEALD interventions in a PCN environment in Alberta, Canada, we showed that the *on-off* design resulted in balanced allocation of patient groups in terms of important clinical characteristics. PCN staff reported valuing research and a willingness to implement the trials. PCN staff were initially aware of the features of the *on-off* design and did not anticipate challenges with its implementation in the primary care setting. However, a juxtaposition of the theoretical perspective of *on-off* design and the perspectives of the health workers during and after implementing the design in the PCN revealed a contrast; that is, perceptions and experiences of healthcare providers implementing the design showed an overall dissatisfaction with it. Specifically, providers described the process of allocating patients to the *off* group as unethical and immoral and in direct conflict with their professional principle of providing care for all. The degree of dissatisfaction seemed exacerbated by: 1) the patient population involved (e.g., patient population viewed as high-risk (e.g., depressed or suicidal), 2) conducting assessments without taking action (e.g., administering the PHQ-9 and not acting on the results), and 3) the (non-blinded) allocation process. It is noteworthy, however, that all of the concerns expressed by PCN staff would be equally applicable to a patient-level or even cluster-randomized trial. Alternate designs, such as the stepped wedge design [28] with a staggered rollout of a new treatment strategy, may not evoke the same perceptions.

Interestingly, the implementation of a new model of care through a controlled trial design revealed an implicit perception of healthcare providers that previous “usual care” was less adequate than the intervention. Reinforcing this perception is the health workers suggestion that patients could act as their own controls and everyone receive the intervention. PCN administrators and healthcare providers were informed that the interventions were proven effective in similar settings and were provided with published evidence during information sessions and training. This may have contributed to the perception that the interventions were more effective than usual care. Therefore, further research is required to explore healthcare providers’ experiences in implementing research designs in the primary care setting, including facilitators and barriers, and regarding fidelity.

Given the potential for conflict with professional values with research designs, like the *on-off* design, it may be necessary to provide resources to hire research assistants to allocate patients and collect data related to the study and have the healthcare providers deliver the intervention to the “*on*” group only. A greater emphasis on understanding the purpose of research in the training of healthcare professionals may help to alleviate some of these issues. It may also be useful for researchers to consult with administrators and healthcare providers tasked with implementation prior to implementing studies to discuss the patient populations involved, types of assessments to be conducted, and the patient allocation process to determine if modifications are necessary, like using research assistants for data collection, to better align with professional values. This consultation process may achieve greater support from partners to adhere to the research design if and when challenges arise.

## Conclusions

In summary, the *on-off* design offers a practical alternative to the randomized allocation for assessing the effectiveness of interventions in the primary care setting and appears to generate evidence that is reasonably valid as well as applicable and relevant to healthcare providers. In addition, this research design may be acceptable to agencies including governmental and non-governmental agencies who may not fund RCTs or research per se. However, it is necessary for research designs to be selected based on the purpose of the study as well as the appropriateness of the design to the setting, including professional values and the patient population involved. Without doing so the connection between the research purpose and the influence or impact on policy and practice may continue to appear contrary to professional training.

## Abbreviations

ABCD: Alberta’s Caring for Diabetes
BMI: body mass index
CDM: chronic disease manager
CM: care manager
ED: executive director
EPOC: Effective Practice and Organization of Care
ES: exercise specialist
GOF: goodness of fit
HbA1_c_: glycosylated hemoglobin
HDL: high-density lipoprotein
LDL: low-density lipoprotein
NCT: non-randomized controlled trial
PCN: primary care network
PHQ-9: Patient Health Questionnaire 9-item
RCT: randomized controlled trial
FP: Family Physician
GI: Glycemic Index
